# Mechanisms underlying treatment effects of vestibular noise stimulation on postural instability in patients with bilateral vestibulopathy

**DOI:** 10.1007/s00415-023-12085-3

**Published:** 2023-11-16

**Authors:** Max Wuehr, Josefine Eder, Silvy Kellerer, Tamara Amberger, Klaus Jahn

**Affiliations:** 1 German Center for Vertigo and Balance Disorders, Ludwig-Maximilians-University, Marchioninistrasse 15, 81377 Munich, Germany; 2 Schön Klinik Bad Aibling, Bad Aibling, Germany

**Keywords:** Bilateral vestibulopathy, Galvanic vestibular stimulation, Stochastic resonance, Balance, Body sway

## Abstract

**Background:**

Previous studies indicate that imbalance in patients with bilateral vestibulopathy (BVP) may be reduced by treatment with low-intensity noisy galvanic vestibular stimulation (nGVS).

**Objective:**

To elucidate the potential mechanisms underlying this therapeutic effect. In particular, we determined whether nGVS-induced balance improvements in patients are compatible with stochastic resonance (SR)—a mechanism by which weak noise stimulation can paradoxically enhance sensory signal processing.

**Methods:**

Effects of nGVS of varying intensities (0–0.7 mA) on body sway were examined in 19 patients with BVP standing with eye closed on a posturographic force plate. We assumed a bell-shaped response curve with maximal sway reductions at intermediate nGVS intensities to be indicative of SR. An established SR curve model was fitted on individual patient outcomes, and three experienced human raters had to judge whether responses to nGVS were consistent with the exhibition of SR.

**Results:**

nGVS-induced reductions of body sway compatible with SR were found in 12 patients (63%) with optimal improvements of 31 ± 21%. In 10 patients (53%), nGVS-induced sway reductions exceeded the minimally important clinical difference (optimal improvement: 35 ± 21%), indicative of strong SR. This beneficial effect was more likely in patients with severe vestibular loss (i.e. lower video head impulse test gain; *R* = 0.663; *p* = 0.002) and considerable postural imbalance (baseline body sway; *R* = 0.616; *p* = 0.005).

**Conclusions:**

More than half of the assessed patients showed robust improvements in postural balance compatible with SR when treated with nGVS. In particular, patients with a higher burden of disease may benefit from the non-invasive and well-tolerated treatment with nGVS.

## Introduction

Chronic postural instability during standing and walking, which aggravates in darkness and on uneven ground, is a cardinal symptom in patients with bilateral vestibulopathy (BVP) [1–3]. Postural deficits may partially ameliorate as patients adapt behavioural strategies that recalibrate multisensory balance and locomotion control [4–6]. However, deficits typically do not dissipate over time [4, 7], which often results in long-term functional impairment and puts patients at an increased risk for recurrent falling [8, 9].

Therapy of postural deficits in BVP is currently primarily based on vestibular rehabilitation that facilitates behavioural adaptions to chronic vestibular hypofunction [6, 10, 11]. However, treatment by physical therapy yields, if any, only partial compensation for lost vestibular feedback [12]. Patients who cannot compensate centrally via vestibular rehabilitation may in the future benefit from the implantation of a vestibular prosthesis, which has shown first promising effects on postural and other BVP-related symptoms in selected patients [13, 14]. However, benefits of an invasive vestibular implant have to be weighed against the risks and cost associated to surgery.

Based on the fact that a majority of patients with BVP retain residual vestibular excitability and function [15, 16], attempts have been made to augment residual vestibular excitability of patients by means of a non-invasive, low-intensity noise stimulation of the vestibular endorgans using noisy galvanic vestibular stimulation (nGVS) [17–19]. Treatment with nGVS has been shown to not only facilitate residual vestibular perceptual and sensorimotor function in patients with BVP [20, 21] but to also stabilise their impaired balance capability during static and dynamic postural tasks [18, 22–26]. As of now, the underlying mode of action of nGVS therapy in patients with BVP is poorly understood. Furthermore, as previous studies consistently observed that not all patients equally respond to stimulation and show a clinically meaningful improvement under treatment [18, 20–26], patient-related factors that may promote or prevent individual treatment success have to be elucidated.

To overcome these deficits, the current study examined individual treatment effects of nGVS on static postural stability in patients with BVP across a broad range of stimulation intensities. In accordance to previous studies, we hypothesised that nGVS modulates vestibular balance function by means of stochastic resonance (SR)—a phenomenon according to which (pathologically increased) thresholds for sensory information processing can be lowered by application of an appropriate amount of low-intensity sensory noise [27, 28]. Exhibition of SR is typically characterised by a noise-induced modulation of the system's output that follows a bell-shaped performance curve with increasing noise intensity, which peaks at a specific intermediate level of noise intensity that optimally facilitates signal transfer within the system. We applied different previously established quantitative and qualitative criteria [29–32] to determine on an individual patient level whether nGVS-induced modulations in balance of patients with BVP are compatible with the exhibition of SR (i.e. display a bell-shaped response curve) or follow other response dynamics. We further examined whether disease-related (aetiology, severity of symptoms, etc.) or demographic factors (age, gender, etc.) may be related to the presence or the absence of treatment responses in individual patients.

## Materials and methods

### Participants

Nineteen patients with BVP (age 59.9 ± 15.4 years, 9 females) participated in the study and provided written informed consent prior to inclusion. Detailed patient characteristics are provided in Table 1. All patients showed a clinically proven deficit, i.e. a bilateral pathological video head impulse test (vHIT, horizontal gain < 0.6) and/or bilateral reduced or absent caloric responses (sum of maximal peak velocities of the slow-phase nystagmus with cold and warm water < 6 °/s) [33]. Fifteen age-matched healthy controls (age 57.7 ± 4.7 years, 7 females) were included in the study to establish normative data. All participants gave written informed consent prior to study inclusion.

**Table 1:** Clinical characteristics and global stimulation effects of patients

Patient	Sex	Age	Aetiology	Caloric response, deg/s^a^		vHIT gain		Optimal nGVS, mA	Exhibition of SR
				Left	Right	Left	Right		
P1	M	62	Idiopathic	–	–	0.33	0.58	0.2	Weak
P2	F	50	Idiopathic	4.4	1.3	0.18	0.16	0.1	Strong
P3	M	82	Idiopathic	2.3	1.3	0.50	0.26	0.5	Strong
P4	M	37	Idiopathic	13.9	2.8	0.53	0.15	-	None
P5	F	82	Infectious	8.9	21.1	0.51	0.56	–	None
P6	M	62	Idiopathic	–	–	0.64	0.56	0.3	Weak
P7	F	65	Idiopathic	3.8	1.1	0.66	0.34	–	None
P8	F	32	Autoimmune	4.0	1.5	0.15	0.27	0.3	Strong
P9	F	69	Neuro-degenerative	5.0	3.5	0.29	0.58	0.5	Strong
P10	M	61	Neuro-degenerative	0.9	0.4	0.00	0.02	0.1	Strong
P11	M	58	Idiopathic	13.6	4.9	0.26	0.57	0.3	Strong
P12	F	81	Idiopathic	5.5	3.9	0.45	0.56	0.7	None
P13	F	54	Neuro-degenerative	3.6	2.3	0.22	0.15	0.4	Strong
P14	M	69	Ototoxic	–	–	0.39	0.54	0.7	None
P15	M	53	Idiopathic	6.9	1.0	0.73	0.78	–	None
P16	M	60	Ototoxic	3.0	2.0	0.10	0.10	0.4	Strong
P17	F	28	Autoimmune	0.9	0.9	0.27	0.47	0.4	Strong
P18	M	72	Ototoxic	1.2	0.8	0.28	0.33	0.7	None
P19	F	61	Idiopathic	4.2	1.4	0.30	0.17	0.1	Strong

*vHIT* video head impulse test, *nGVS* noisy galvanic vestibular stimulation

^a^Sum of maximal slow-phase eye velocity during warm and cold caloric irrigation

### Galvanic vestibular stimulation

Vestibular noise stimulation (i.e. nGVS) was applied via a pair of 4.0 cm × 6.0 cm Ag–AgCl electrodes attached bilaterally over the left and right mastoid process. Zero-mean Gaussian white noise stimulation with a frequency range of 0–30 Hz and varying peak amplitudes of 0–0.7 mA was delivered by a mobile constant current stimulator (neuroConn®, Illmenau, Germany).

### Experimental procedures

**Fig. 1:**
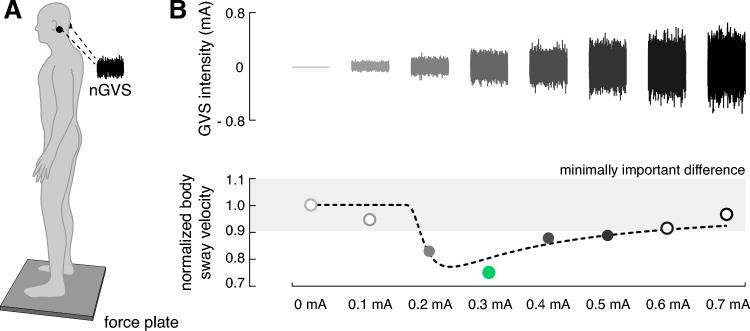
Experimental setup and procedures. **A** Effects of noisy galvanic vestibular stimulation (nGVS) on static balance in patients were measured on a posturographic force plate. Velocity of body sway was calculated from the resultant center-of-pressure trajectories. **B** Exemplary modulation of body sway (simulated data, lower panel) across the administered nGVS intensities (upper panel) that follows a bell-shaped performance curve indicative of the presence of stochastic resonance (model fit: dashed line). Filled dots indicate body sway reductions greater than the minimally important difference (grey area). The green filled dot indicates the optimal reduction of body sway at a particular nGVS level

Body sway was recorded for 30 s on a posturographic force plate (Kistler, 9261A, Kistler Group, Winterthur, Switzerland) at 40 Hz whilst patients were standing with their eyes closed (Fig. 1A). This procedure was repeated eight times, whilst patients were stimulated with a different amplitude of nGVS (ranging from 0 to 0.7 mA, in a randomised order) in each trial. Patients were blinded to the exact stimulation order. Between trials, patients were given a short break to recover.

### Data and statistical analysis

For each stance trial, mean sway velocity was calculated as the primary output measure based on the recorded radial centre-of-pressure (CoP) trajectory using the formula $SV = 1/T \times {\sum }_{i}\left|{r}_{i+1}-{r}_{i}\right|, [\mathrm{mm}/\mathrm{s}]$, where $T$ is the total trial duration (i.e. 30 s) and ${r}_{i}$ is the radial CoP distance of the $ith$ sample. For further analysis, sway velocity measures from 8 stance trials were normalised to sway velocity obtained during 0 mA stimulation (i.e. baseline condition).

To determine whether SR-like dynamics were present in the balance responses of patients to varying nGVS levels, we tested three increasingly rigorous criteria built on one another: (1) The first criterion tested whether body sway of patients improved for at least one particular nGVS level compared to baseline condition (i.e. 0 mA nGVS). (2) The second criterion was based on a visual inspection of response dynamics of body sway across increasing nGVS level by three experienced human raters (i.e. MW, JE, and KJ). Each rater had to evaluate whether (in addition to the fulfilment of the first criterion) nGVS-amplitude-dependent changes of body sway in individual patients were further compatible with a bell-shaped response curve with improvements of performance at intermediate stimulation intensities that is indicative of the presence of SR. For this evaluation, each rater was independently provided with a plot of the normalised nGVS-dependent changes in body sway and a superimposed theoretical SR curve that was fit on the data using a goodness-of-fit statistics [29, 30] (see example Fig. 1B). The applied equation fit represents an adapted version of the originally proposed SR model by Benzi [34], including a piecewise, linear masking effect to model cases where nGVS effects at high amplitudes may have detrimental effects on the performance metric [35]. The criterion was met if at least two of the raters identified the presence of SR-like dynamics. (3) The third criterion additionally evaluated whether improvements at intermediate nGVS levels were greater than the minimal clinically important difference (MCID; defined as half the standard deviation for normative data [36]) for changes in body sway velocity. MCID for sway velocity was 2.3 mm/s calculated based on the posturographic recordings of the 15 age-matched healthy individuals standing with eyes closed for 30 s.

Based on the three criteria, patients were classified as showing solely optimal improvement and no SR (criterion 1), exhibiting weak SR (criteria 1 & 2) or showing strong SR (criterion 1, 2, & 3). Potential correlations between SR classification and age, gender, aetiology, vHIT gain, caloric response, and baseline body sway were analysed using Spearman's rank correlation. Results were considered significant at *p* < 0.05. Statistical analysis was performed using SPSS (Version 26.0, IBM Corp., USA).

## Results

Application of nGVS at intensities ranging from 0.1 to 0.7 mA was well tolerated and did not cause apparent disequilibrium in any of the examined patients. In the first step of analysis, we evaluated whether body sway velocity was decreased by at least one particular nGVS intensity compared to sham stimulation (i.e. nGVS at 0 mA). This criterion was met by 15 patients (79%) with an optimal improvement magnitude of in average 29% (range 4–69%) at an average intensity of 0.4 mA (range: 0.1–0.7 mA).

**Fig. 2:**
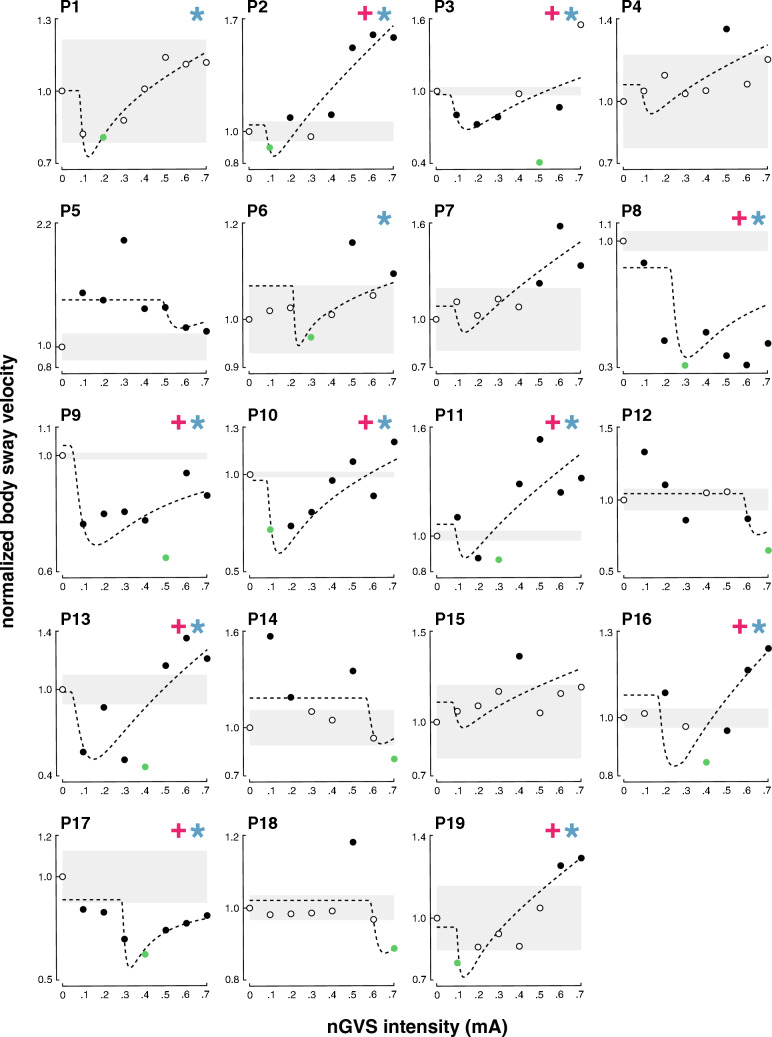
Individual effects of low-intensity vestibular noise stimulation on static balance. Normalised body sway responses to noisy galvanic vestibular stimulation (nGVS) are plotted against the administered nGVS levels for each individual patient. Dashed lines represent the stochastic resonance (SR) model fits. Black filled dots indicate body sway modulations greater than the minimally important clinical difference (grey area). Green filled dots indicate optimal reductions of body sway at particular nGVS levels. Blue asterisks denote those patients that exhibit SR-like responses according to three human judges (weak SR). Pink crosses denote those patients that additionally show clinically meaningful improvement of body sway (strong SR)

**Fig. 3:**
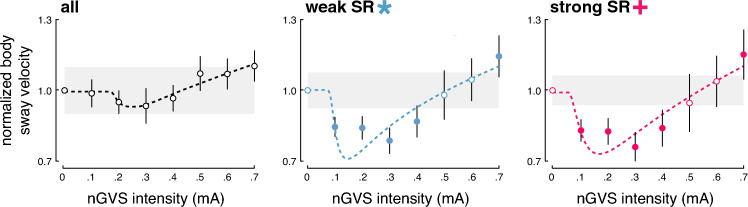
Group average effects of low-intensity vestibular noise stimulation on static balance. Group average normalised body sway responses (mean ± SEM) to noisy galvanic vestibular stimulation (nGVS) are plotted for each of the administered nGVS levels for all patients (left panel), those patients exhibiting weak stochastic resonance (SR; middle panel), and those exhibiting strong SR (right panel). Filled dots indicate body sway modulations greater than the minimally important clinical difference (grey area)

In the second step, an established SR model was fit to the individual modulations of body sway velocity in dependence of nGVS intensity (Fig. 2). Three experts were asked to independently rate for each patient by visual inspection of individual sway velocity modulations and corresponding model fits whether body sway responses follow a bell-shaped performance curve or not. Based on their judgments, SR-like treatment responses to nGVS were present in 12 patients (63%) with optimal improvements of 31% (range 4–69%) at an average intensity of 0.3 mA (range: 0.1–0.5 mA). Analogous bell-shaped performance modulations with optimal improvement at intermediate noise intensities were found on the group average response level of these patients (Fig. 3). In the remaining patients (37%), body sway velocity either randomly fluctuated (3 patients) or was generally increased (4 patients) across the range of tested nGVS intensities.

We subsequently identified those patients that in addition to SR-like response dynamics showed a clinically meaningful improvement of static balance (i.e. a reduction of body sway velocity greater than the MCID, Fig. 2). This criterion for the exhibition of strong SR was met by 10 patients (53%) with an average optimal improvement of 35% (range 10–69%) at an average intensity of 0.3 mA (range: 0.1–0.5 mA). Considerable SR-like performance improvements were also apparent on the group average level of patients exhibiting strong SR (Fig. 3).

In the final step, we explored demographic and disease-related factors that may potentially promote or hamper the exhibition of weak or strong SR in response to nGVS treatment. Correlation analysis revealed a positive association between baseline levels of static body sway (i.e. sway velocity assessed during nGVS at 0 mA; *R* = 0.616; *p* = 0.005) and a negative association with the vHIT gain assessed during clinical examination (*R* = − 0.663; *p* = 0.002). Hence, patients with profound postural impairments at baseline and a significant vestibulo-ocular reflex deficit were more likely to exhibit SR-like balance improvements at clinically meaningful effects sizes under treatment with nGVS.

## Discussion

There is increasing evidence that postural symptoms in patients with BVP may ameliorate in response to a non-invasive, low-intensity noise stimulation of the vestibular endorgans (i.e. nGVS) [18, 22–26]. Albeit the mode of action underlying this treatment effect was repeatedly attributed to SR in vestibular sensorimotor and/or perceptual pathways, previous studies failed to provide sufficient evidence for the latter assumption. The reason for this is that these studies typically limited the application and/or analysis of treatment outcomes to one particular noise intensity and could thus not determine whether postural responses follow a SR-like bell-shaped response curve with increasing noise intensity. Since a better understanding of the treatment principle underlying nGVS is important for future therapeutic applications, we here explicitly evaluated nGVS treatment effects to nGVS across a broad range of noise intensities to determine (1) whether nGVS-induced modulations of postural imbalance in individual patients are compatible with the exhibition of SR and to further identify (2) demographic and/or disease-related factors that may qualify patients to particularly benefit from treatment with nGVS.

Our analysis revealed that postural responses in about two thirds of patients closely followed a bell-shape performance curve with optimal balance improvements at intermediate noise intensities—a response rate that is considerably higher than previously reported in young healthy individuals where nGVS-induced balance responses compatible with SR were only rarely observed [30]. Static balance of patients was optimally stabilised at an average intensity of 0.3 mA (range: 0.1 to 0.5 mA), which is compatible to previous reports on nGVS-induced SR in healthy individuals and other clinical cohorts [29, 31] and approximates 60% of the estimated detection threshold of vestibular afferent responses to GVS [37]. We further found that at least half of the patients showed nGVS-induced balance improvements at clinically meaningful effect sizes. In the remaining third of patients, nGVS-induced balance responses did not exhibit SR-like response dynamics. In some of these, balance responses did not show any systematic dependency on nGVS and thus likely reflect variations in the performance metric (i.e. body sway) rather than any therapeutic effect. In others, nGVS treatment degraded balance performance irrespective of stimulation intensity, which might indicate a general intolerance to low-intensity vestibular noise stimulation.

We further explored potential demographic and/or disease-related factors that may influence nGVS treatment response in individual patients. We found that the integrity of vestibulospinal and vestibulo-ocular reflex function was associated with the presence or absence of stimulation benefits. Accordingly, patients with greater postural instability during visual withdrawal—a proxy for impairment of vestibular (and proprioceptive) balance regulation—were more likely to exhibit SR-like balance improvements at clinically meaningful effects sizes under treatment with nGVS. Analogously, we found that patients with a lower gain during vHIT assessment—a proxy for the impairment of vestibulo-ocular reflex function—showed greater benefits from nGVS treatment. This suggests that patients with residual but severely compromised peripheral vestibular function may particularly benefit from treatment with low-intensity vestibular noise stimulation. Similar associations between nGVS treatment response and the capacity or integrity of vestibular function were found in young and healthy elderly adults [38, 39].

Taken together with previous evidence from studies in vestibular animal models and humans, the current results shed light on the presumable mode of action underlying nGVS treatment effects on static balance. Previous studies in frog and chicken demonstrated that low-intensity noise exerted on the vestibular endorgans induces SR-like improvements of vestibular signal transfer at the level of vestibular hair cells and primary vestibular afferents [40, 41]. Subsequent studies in humans indicate that noise-induced improvements in signal processing at the vestibular periphery are conveyed to centrally mediated vestibulospinal and vestibular perceptual functions. Accordingly, both healthy individuals and patients with BVP exhibit a SR-like sensitisation of vestibular motion perception in response to nGVS treatment [21, 29, 42, 43]. Analogously, nGVS was shown to induce SR-like enhancement of vestibulospinal responses in both cohorts [20, 44]. Both of these effects are likely to contribute to the observed SR-like stabilisation of postural imbalance in patients with BVP. Accordingly, previous evidence indicates that vestibular balance control is not confined to vestibulospinal reflex control but also involves the perceptual registration of head and body in space [45, 46]. Our observations further suggest, that nGVS-induced enhancements at the vestibular reflex and perceptual level only manifest in a clinically meaningful postural stabilisation in individuals with significantly compromised balance performance at baseline.

In conclusion, we found evidence that low-intensity noise stimulation ameliorates postural imbalance in about two thirds of the assessed patients with BVP. In particular, patients with severe impairments of peripheral vestibular function are likely to show balanced improvements at clinically meaningful effects sizes under treatment. nGVS-induced balance improvements in these patients are further consistent with the exhibition of SR in vestibular sensorimotor and perceptual pathways. Future studies are required to investigate whether nGVS may analogously target other BVP-related impairments in gaze stabilisation and spatial cognition.

## Data Availability

The datasets used and/or analysed during the current study will be available from the corresponding author upon reasonable request.
